# Glucagon-like peptide 1 receptor agonists and cancer risk: advancing precision medicine through mechanistic understanding and clinical evidence

**DOI:** 10.1186/s40364-025-00765-3

**Published:** 2025-03-27

**Authors:** Anqi Lin, Yanxi Ding, Zhengrui Li, Aimin Jiang, Zaoqu Liu, Hank Z. H. Wong, Quan Cheng, Jian Zhang, Peng Luo

**Affiliations:** 1 Donghai County People’s Hospital - Jiangnan University Smart Healthcare Joint Laboratory, Donghai County People’s Hospital (Affiliated Kangda College of Nanjing Medical University), Lianyungang, Jiangsu Province 222000 China; 2 Department of Oncology, Zhujiang Hospital, Southern Medical University, Guangzhou, 510282 Guangdong China; 3 The Second School of Clinical Medicine, Southern Medical University, Guangzhou, Guangdong 510515 China; 4 Department of Oral and Cranio-Maxillofacial Surgery, Shanghai Ninth People’s Hospital, College of Stomatology, Shanghai Jiao Tong University School of Medicine, National Clinical Research Center for Oral Diseases, Shanghai Key Laboratory of Stomatology and Shanghai Research Institute of Stomatology, Shanghai, China; 5 Department of Urology, Changhai Hospital, Naval Medical University (Second Military Medical University), Shanghai, China; 6 Institute of Basic Medical Sciences, Chinese Academy of Medical Sciences and Peking Union Medical College, Beijing, 100730 China; 7 Li Ka Shing, Faculty of Medicine, The University of Hong Kong, Hong Kong SAR, China; 8 Department of Neurosurgery, Xiangya Hospital, Central South University, Changsha, Hunan 410008 China; 9 National Clinical Research Center for Geriatric Disorders, Xiangya Hospital, Central South University, Hunan, China

**Keywords:** Glucagon-like peptide-1 receptor agonists, GLP-1-RAs, Precision medicine, Cancer risk

## Abstract

Glucagon-like peptide-1 receptor agonists (GLP-1RAs) have emerged as a primary first-line treatment for type 2 diabetes. This has raised concerns about their impact on cancer risk, spurring extensive research. This review systematically examines the varied effects of GLP-1RAs on the risk of different types of tumors, including overall cancer risk and specific cancers such as thyroid, pancreatic, reproductive system, liver, and colorectal cancers. The potential biological mechanisms underlying their influence on cancer risk are complex, involving metabolic regulation, direct antitumor effects, immune modulation, and epigenetic changes. A systematic comparison with other antidiabetic agents reveals notable differences in their influence on cancer risk across drug classes. Additionally, critical factors that shape the relationship between GLP-1RAs and cancer risk are thoroughly analyzed, including patient demographics, comorbidities, treatment regimens, and lifestyle factors, offering essential insights for developing individualized treatment protocols. Despite significant research progress, critical gaps remain. Future research should prioritize elucidating the molecular mechanisms behind the antitumor effects, refining individualized treatment strategies, investigating early tumor prevention applications, assessing potential benefits for non-diabetic populations, advancing the development of novel therapies, establishing robust safety monitoring frameworks, and building precision medicine decision-making platforms. These efforts aim to establish novel roles for GLP-1RAs in cancer prevention. and treatment, thereby advancing the progress of precision medicine.

## Introduction

Amid the continuously increasing global incidence of diabetes, glucagon-like peptide-1 receptor agonists (GLP-1RAs) have emerged as a cornerstone in type 2 diabetes treatment, demonstrating remarkable efficacy in glycemic control and cardiovascular risk reduction [1, 2]. While their therapeutic benefits are well-established, the relationship between GLP-1RAs and cancer risk has become a critical focus of clinical investigation. Current evidence from large-scale randomized controlled trials, observational studies, and real-world data presents a complex picture of this relationship. Randomized controlled trials have demonstrated that semaglutide treatment does not increase overall cancer risk [3], while observational studies have revealed varying effects across different cancer types [4, 5]. For instance, retrospective analyses have suggested potential associations with thyroid cancer risk during long-term use [4], whereas studies of pancreatic cancer have yielded contrasting results [6–8].

The biological impact of GLP-1RAs on cancer risk operates through multiple mechanistic pathways, including direct effects on tumor cell metabolism and proliferation, modulation of immune responses in the tumor microenvironment, and regulation of inflammatory signaling networks (9–11). The clinical effects appear to be influenced by several key factors, including patient characteristics (age, gender, ethnicity), concurrent medical conditions (particularly diabetes and obesity), and treatment parameters (drug type, dosage, duration) [9, 10]. This variability in outcomes likely stems from differences in study design, population characteristics, and follow-up duration.

The complex interplay between diabetes and cancer risk presents additional challenges in understanding the role of GLP-1RAs. Diabetes itself is associated with increased risk of various cancers, potentially through mechanisms involving chronic inflammation, insulin resistance, and metabolic dysregulation [11, 12]. Different diabetes medications demonstrate varying effects on cancer risk, with some showing protective effects while others may potentially increase risk in certain populations [10, 13, 14].

Despite significant advances in understanding GLP-1RAs' mechanisms of action and their application in diabetes treatment, several critical knowledge gaps persist. Current research findings show considerable variation across different cancer types and patient populations [4, 6], highlighting the need for more targeted investigation. The molecular mechanisms underlying GLP-1RAs' regulation of tumor development and progression remain incompletely understood [13], limiting clinicians' ability to accurately assess patient risks and implement targeted interventions. Furthermore, systematic clinical evidence regarding the long-term impact of GLP-1RAs on cancer risk is lacking, creating challenges for assessing the long-term safety of these treatments. In the context of precision medicine, developing optimized GLP-1RAs treatment plans based on individual cancer risk profiles while balancing diabetes management remains an urgent clinical challenge.

In recent years, GLP-1RAs have made significant advancements in the treatment of diabetes; however, their association with cancer risk remains a subject of ongoing debate. This review seeks to systematically elucidate the relationship between GLP-1RAs and cancer risk, along with the potential underlying mechanisms involved. The article first reviews the heterogeneous effects of GLP-1RAs on various cancer risks and their supporting clinical evidence. It then investigates the underlying mechanisms of action of GLP-1RAs, focusing on: 1) metabolic regulatory pathways; 2) direct modulation of cell proliferation and apoptosis; and 3) regulation of the immune system. Furthermore, the review explores key clinical factors influencing cancer risk associated with GLP-1RAs, such as patient population characteristics and medication regimens. It also presents a comparative analysis of the differences in cancer risk between GLP-1RAs and other antidiabetic medications. Additionally, the review discusses the prospective applications of GLP-1RAs in both cancer prevention and treatment.

## Differential effects of GLP-1RAs on the risk of diverse tumor types

Currently, several GLP-1RAs have been approved for the treatment of type 2 diabetes (T2DM) and/or obesity, and emerging evidence suggests that these agents may influence cancer risk. Accumulating evidence demonstrates that GLP-1RAs exert heterogeneous effects across different tumor types (Table 1). According to Table 1, comprehensive experimental and observational studies reveal that GLP-1RAs do not elevate overall cancer risk, while preclinical investigations have demonstrated multiple anti-tumor mechanisms, including proliferation inhibition, apoptosis induction, and metastasis suppression. Nevertheless, the risk profiles exhibit marked heterogeneity among different cancer types. Regarding thyroid and pancreatic cancers, contemporary evidence remains inconclusive, with conflicting studies reporting either elevated risk or no significant correlation. In reproductive system malignancies, GLP-1RAs predominantly exhibit neutral or protective effects, notably in prostate cancer, where preclinical studies have demonstrated anti-proliferative activities. Significantly, GLP-1RAs demonstrate favorable protective effects against hepatocellular and prostate cancer. In other malignancies, including pulmonary and cutaneous neoplasms, no significant elevation in risk has been documented, with emerging evidence indicating potential protective properties. These heterogeneous responses are modulated by multiple factors, including tumor classification, study methodology, treatment duration, and patient-specific parameters (Table 1). Systematic reviews have revealed that GLP-1RAs exert bidirectional modulatory effects on cancer risk, with outcomes varying depending on cancer type, patient characteristics, and study methodology. Large-scale clinical trials have demonstrated that GLP-1RAs predominantly exhibit neutral or suppressive effects on overall cancer risk, with semaglutide showing no elevated cancer incidence [14], while significant reductions have been observed in specific malignancies, particularly liver cancers and prostate cancers [13, 15]. The observed protective effects are mediated through multiple molecular mechanisms, including enhanced metabolic regulation, direct anti-proliferative actions, and augmented immune surveillance [16, 17]. However, observational studies have identified potential risk-amplifying effects in specific clinical contexts. Specifically, prolonged GLP-1RA administration (1–3 years or longer) has been associated with elevated thyroid cancer risk in certain populations [4], while high concentrations of liraglutide (100 nM) have been demonstrated to promote breast cancer progression via the NOX4/ROS/VEGF signaling pathway [18]. These divergent effects appear to be modulated by multiple factors, including treatment duration, drug concentration, cancer type, and patient-specific parameters such as genetic background and comorbidities [9, 19].

**Table 1:** Association between GLP-1 receptor agonists (GLP-1RAs) and risk of specific cancer types

Cancer type	Study design	Main findings	References
Overall Cancer Risk	Experimental Study	GLP-1RAs do not increase overall cancer risk	3
	Observational Study	Semaglutide does not elevate overall cancer risk	14
	Preclinical Studies	GLP-1RAs Show anti-tumor properties: inhibit proliferation, induce apoptosis, suppress metastasis	13
Thyroid Cancer	Observational Study, Meta-analysis, Experimental Study	Contradictory results; some studies show increased risk with certain GLP-1RAs, while others find no significant association	1, 2, 4, 5, 9, 20–24
Pancreatic Cancer	Meta-analysis, Observational Study	Contradictory results; some reports suggest increased risk, particularly with exenatide, while others indicate no association	6, 7, 8, 25–29
Breast Cancer	Experimental Study, Observational Study	Generally not increased; some GLP-1RAs reduce risk, though liraglutide shows increased cell proliferation in vitro	18, 31–34, 36, 38
Prostate Cancer	Preclinical Studies	GLP-1RAs may inhibit cell proliferation, suggesting protective effects, especially with exendin-4 and liraglutide	17, 36, 37
Endometrial and Ovarian Cancer	Preclinical Studies	GLP-1RAs show antitumor activity in endometrial cancer; no increased risk of ovarian cancer, with potential protective benefits	38–41
Liver Cancer	Preclinical Studies	GLP-1RAs, particularly liraglutide, exhibit prevention and reduction of liver cancer risk in diabetic and NASH models	42–44
Colorectal Cancer	Observational Study	Reduced risk with prolonged use, although not recommended as first-line therapy in high-risk patients	38, 45–47
Other Cancers (Lung, Skin)	Observational Study	No significant increased risk; potential protective effect on lung cancer confirmed through stratified analyses	19, 48, 49

*Abbreviations*: *GLP-1RAs* glucagon-like peptide-1 receptor agonists, *NASH* nonalcoholic steatohepatitis, -: does not elevate risk and may be potentially protective, ↓: risk reduction, ?: contradictory results

### The effects of GLP-1RAs on thyroid cancer risk

Multiple retrospective observational studies and spontaneous case reports suggest a possible association between GLP-1RAs and an elevated risk of thyroid cancer [20, 21]. Furthermore, a meta-analysis suggested a possible association between GLP-1RAs therapy and an increased relative risk of thyroid cancer in clinical trials, with liraglutide, exenatide, and dulaglutide potentially elevating cancer risk in patients with type 2 diabetes [9]. The clinical evidence by Bezin et al. indicated that GLP-1RAs treatment might increase the risk of thyroid cancer, with the risk demonstrating an upward trend with prolonged usage of the medication (1–3 years and longer) [4]. Compared to patients treated with sodium-dependent glucose transporters 2 (SGLT2) inhibitors as monotherapy, those receiving GLP-1RAs for type 2 diabetes exhibited a significantly increased risk of thyroid hyperplasia and thyroid cancer [22]. Yet there are many more studies explaining that the use of GLP-1RAs may not be associated with an increased risk of thyroid cancer. In animal experiments on such drugs, studies on rodents have found that the GLP-1RAs may promote the development of medullary thyroid carcinoma, but this effect has not been observed in non-human primates [23]. A large multinational cohort study spanning three countries, alongside another randomized controlled trial, both concluded that GLP-1RAs usage was not associated with a significant increase in thyroid cancer risk [2, 5]. Additionally, another robust randomized controlled trial found no association between semaglutide use and an increased risk of thyroid cancer [1]. The heterogeneity of these findings may be attributed to variations in study design, population characteristics, and the types of drug formulations used [9]. Although the relationship between GLP-1RAs and thyroid cancer risk remains uncertain, caution is advised when prescribing GLP-1RAs in individuals with a history of thyroid cancer or in those with higher risk [24].

### The effects of GLP-1RAs on pancreatic cancer risk

The relationship between GLP-1RA administration and pancreatic cancer risk remains a subject of significant debate in contemporary research. Pharmacovigilance studies indicate that GLP-1RAs, with the exception of albiglutide, demonstrate potential associations with pancreatic cancer risk, particularly evident in exenatide-treated patients who exhibit elevated cancer incidence rates. It is well established that patients with diabetes demonstrate higher susceptibility to pancreatic cancer and pancreatitis compared to non-diabetic individuals, and theoretically, sustained GLP-1 receptor activation induced by GLP-1RA therapy may potentially elevate pancreatic cancer risk [8]. Clinical evidence, encompassing trials, epidemiological investigations, and findings from animal toxicology and histological studies, has revealed potential associations between GLP-1RAs and the risk of neuroendocrine tumors and exocrine pancreatic dysplasia, with some evidence suggesting an increased risk of pancreatic cancer [6]. However, meta-analyses incorporating larger cohort studies have demonstrated no significant correlation with elevated pancreatic cancer risk, although the relatively brief duration of these cohort studies may influence these conclusions [25]. Furthermore, multiple additional meta-analyses have consistently shown no significant association between GLP-1RA therapy and pancreatic cancer risk. Given the limitations of sample sizes and individual variability, these findings warrant careful interpretation [8].

Some studies have concluded that the use of GLP-1RAs demonstrates no association with an increased risk of pancreatic cancer [7, 26, 27]. Accumulated clinical evidence suggests that GLP-1RAs not only show no increase in pancreatic cancer risk among diabetic patients but potentially confer a protective effect [26]. Furthermore, liraglutide exhibits potential pancreatic cancer risk-reducing properties and demonstrates efficacy in enhancing the chemosensitivity of pancreatic cancer cells to specific therapeutic agents [8, 28, 29]. Notably, a clinical study demonstrated that T2DM patients receiving insulin therapy experienced significantly higher rates of pancreatitis compared to those treated with GLP-1RAs, and notably, no pancreatitis cases have been reported in clinical trials of the novel GLP-1RA Cotadutide, indicating that a comprehensive risk assessment of GLP-1RAs for pancreatic cancer remains premature pending additional data on these novel medications [8]. While multiple clinical trials and population-based studies have established the safety profile of GLP-1RAs, continued vigilance regarding the association between GLP-1RA therapy and pancreatic cancer risk remains imperative in clinical practice, given the potential for low-grade chronic pancreatitis to induce pancreatic cancer.

### The effects of GLP-1RAs on breast cancer risk

Current evidence indicates that GLP-1RAs not only do not increase the risk of breast cancer but may also be associated with a reduced risk of breast cancer development [30]. Clinical evidence supports the hypothesis that GLP-1RAs exert a direct antitumor effect on hormone receptor-positive breast cancer [31]. Preclinical studies have reported that Exendin-4 (Ex-4) and dulaglutide display inhibitory effects on breast cancer, whereas liraglutide presents a trend of increased breast cancer incidence, with the risk ratio rising with prolonged exposure. At a concentration of 1,000 nM, liraglutide promotes the proliferation of breast cancer cells [18, 32]. However, some preclinical studies have also demonstrated that liraglutide inhibits proliferation and migration in breast cancer cell lines [33]. It is also worth noting that in obese patients with T2DM, GLP-1RA-mediated weight reduction has been associated with the detection rate of breast cancer [34], with the highest incidence observed in patients who experienced more than 10% weight loss following treatment [35].

### The effects of GLP-1RAs on prostate cancer risk

Preclinical studies have demonstrated that GLP-1RAs are capable of inhibiting the proliferation of prostate cancer cells in both in vivo and in vitro experiments, thereby suggesting potential preventative effects [36]. Besides, Ex-4 has been shown to exhibit significant antitumor activity, with its usage being strongly associated with a reduced risk of prostate cancer [17]. Liraglutide has also been shown to significantly reduce the incidence of prostate cancer and improve survival outcomes in diabetic patients diagnosed with prostate cancer [36, 37].

### The effects of GLP-1RAs on endometrial and ovarian cancer risk

Concerning ovarian cancer, current evidence suggests that GLP-1RAs are not associated with an increased risk of ovarian cancer [38]. In one meta-analyses of Ex-4 shown to inhibit the growth of ovarian cancer cells and induce apoptosis [39]. Preclinical studies in mice demonstrate that GLP-1RAs exhibit significant antitumor activity against endometrial cancer cells [40], and their ability to improve metabolism and body weight could offer preventive benefits for individuals at high risk of developing endometrial cancer [41].

### The effects of GLP-1RAs on liver cancer risk

Existing studies indicate that GLP-1RAs not only play a significant role in reducing liver cancer risk but also exhibit notable preventative effects [42]. In vitro and in vivo research has demonstrated that Liraglutide and Exenatide show anti-liver cancer effects by attenuating inflammation and inhibiting the proliferation of liver cancer cells [43]. Related animal studies have further affirmed that Liraglutide can effectively suppress liver cancer progression in mouse models of diabetes and nonalcoholic steatohepatitis (NASH) [44]. However, evidence regarding the association between GLP-1RAs and liver cancer risk is currently limited.

### The effects of GLP-1RAs on colorectal and other cancer risk

Clinical studies have demonstrated that the use of GLP-1RAs can reduce the risk of colorectal cancer, and the protective effect appears to be positively correlated with the duration of therapy. When compared to other antihyperglycemic agents, patients with type 2 diabetes receiving GLP-1RAs therapy exhibit a lower risk of developing colorectal cancer [45]. GLP-1RAs have demonstrated significant anti-colorectal cancer activity [46], with the protective effects increasing in a duration-dependent manner. However, some clinical evidences suggest that GLP-1RA therapy could potentially increase the risk of colorectal cancer in a subset of patients [38]. Based on current evidence, GLP-1RAs are not recommended as first-line therapy for type 2 diabetic patients at high risk for colorectal cancer [47].

Compared to other antidiabetic medications, the use of GLP-1RAs has not been associated with an increased risk of lung cancer [48]; in fact, some clinical evidences suggest it may have a potential protective effect in reducing lung cancer risk. This protective effect has been substantiated through subgroup analyses stratified by histological type, race, gender, and smoking status [19]. In contrast, no correlation was shown between the two in a cohort study on the effect of GLP-1RAs on melanoma or non-melanoma skin cancers risk [49].

## Molecular mechanisms underlying the impact of GLP-1RAs on cancer risk

The regulation of cancer risk by GLP-1RAs involves several biological mechanisms, primarily encompassing metabolic regulation, direct anti-tumor effects, immune modulation, and epigenetic alterations. In terms of metabolic regulation, GLP-1RAs exert their effects through various pathways, including increasing insulin sensitivity, maintaining glucose homeostasis, promoting weight control, and improving lipid metabolism. Regarding direct anti-tumor effects, studies have demonstrated that GLP-1RAs exhibit multiple functions, including inhibiting tumor cell proliferation, inducing apoptosis, and preventing tumor cell migration and invasion. These anti-tumor effects exhibit considerable heterogeneity across various tumor types. In terms of immune modulation, GLP-1RAs demonstrate a bidirectional mechanism: they can enhance anti-tumor immune responses while modulating systemic inflammatory processes. Moreover, GLP-1RAs may affect tumor initiation and progression by modulating microRNA expression patterns and influencing epigenetic modifications.

### The mechanisms underlying the prevention of cancer by GLP-1RAs

#### Improvement in Insulin sensitivity and glycemic control

Chronic hyperglycemia, insulin resistance, and secondary hyperinsulinemia are widely acknowledged as significant risk factors that contribute to tumorigenesis and heightened cancer mortality rates [3, 11]. Hyperinsulinemia and insulin resistance promote tumor growth through various mechanisms: directly by activating the insulin receptor signaling pathway and indirectly by stimulating the insulin-like growth factor (IGF) receptor system, which in turn fosters cellular proliferation, migration, and invasion while inhibiting programmed cell death [11]. Conversely, abnormal blood glucose levels have been shown to elevate cancer risk and decrease survival rates in cancer patients [12, 50]. As GLP-1 analogs, GLP-1RAs effectively regulate blood glucose by inhibiting glucagon secretion, slowing gastric emptying, enhancing insulin sensitivity, and stimulating insulin secretion [51]. Therefore, GLP-1RAs may potentially lower cancer risk by enhancing insulin sensitivity and maintaining glycemic control.

#### Impact on body weight regulation and lipid homeostasis

Increased BMI is linearly and positively associated with the risk of organ-specific tumors [52, 53]. Although the specific molecular mechanisms through which obesity promotes carcinogenesis remain not fully elucidated, epidemiological studies indicate that approximately 4–8% of malignant tumors can be attributed to obesity, and obesity serves as an independent risk factor for poor prognosis in cancer patients [54]. During obesity-induced adipose tissue remodeling, abnormal secretion of proinflammatory factors and adipokines promotes tumor cell proliferation, invasion, and metastasis, accelerating tumor progression. In the obese state, adiponectin levels decrease as leptin levels increase. Leptin exhibits clear pro-carcinogenic effects by promoting systemic inflammatory responses and cellular proliferation, while inhibiting apoptosis and immune surveillance functions, whereas adiponectin exerts the opposite effects [11]. As a representative GLP-1RA drug, liraglutide has been widely recognized as an effective treatment for obesity by suppressing appetite and promoting satiety [51]. By improving the obesity status of patients, reducing weight, optimizing lipid metabolism, and exerting anti-inflammatory effects, GLP-1RAs may also play a positive role in cancer prevention and treatment.

#### Direct regulation of cancer cell proliferation, apoptosis, migration and invasion

**Fig. 1:**
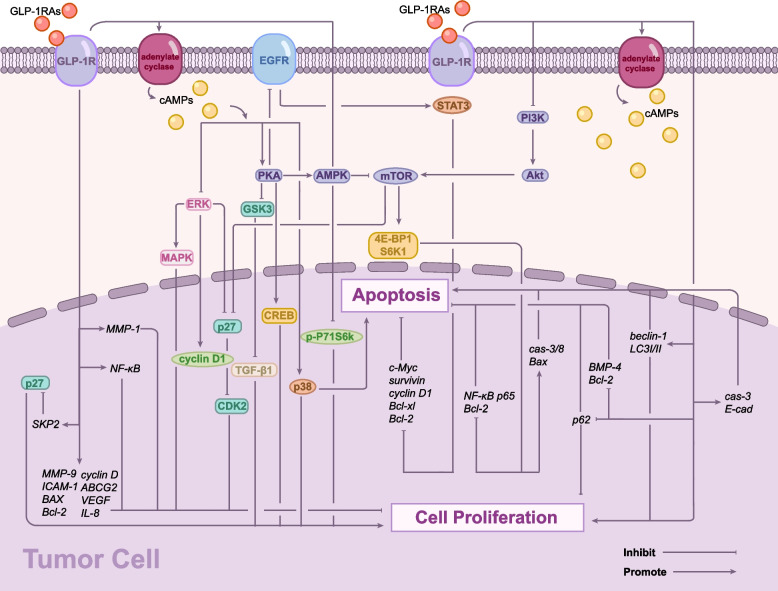
Molecular Mechanisms of GLP-1 Receptor Agonist (GLP-1RA)-Mediated Cancer Cell Growth Inhibition. GLP-1RA binding to GLP-1R triggers multiple signaling cascades that suppress cancer cell proliferation and promote apoptosis. The primary pathways include: (1) cAMP-mediated inhibition of ERK signaling, reducing cyclin D1 expression and DNA replication; (2) PKA-AMPK axis activation, leading to mTOR inhibition and p27-mediated cell cycle arrest; (3) cAMP-dependent activation of p38 pathway promoting apoptosis; (4) PKA-mediated suppression of EGFR-STAT3 signaling, downregulating pro-survival genes; (5) PI3K/Akt/mTOR pathway inhibition, increasing TGF-β1 levels and promoting cell cycle arrest; (6) NF-κB pathway suppression, reducing expression of proliferation-related genes; and (7) Activation of autophagy pathways through regulation of beclin-1, LC3I/II, and p62 expression. Additional mechanisms include SKP2 inhibition, PGR upregulation, and BMP4 downregulation

**Fig. 2:**
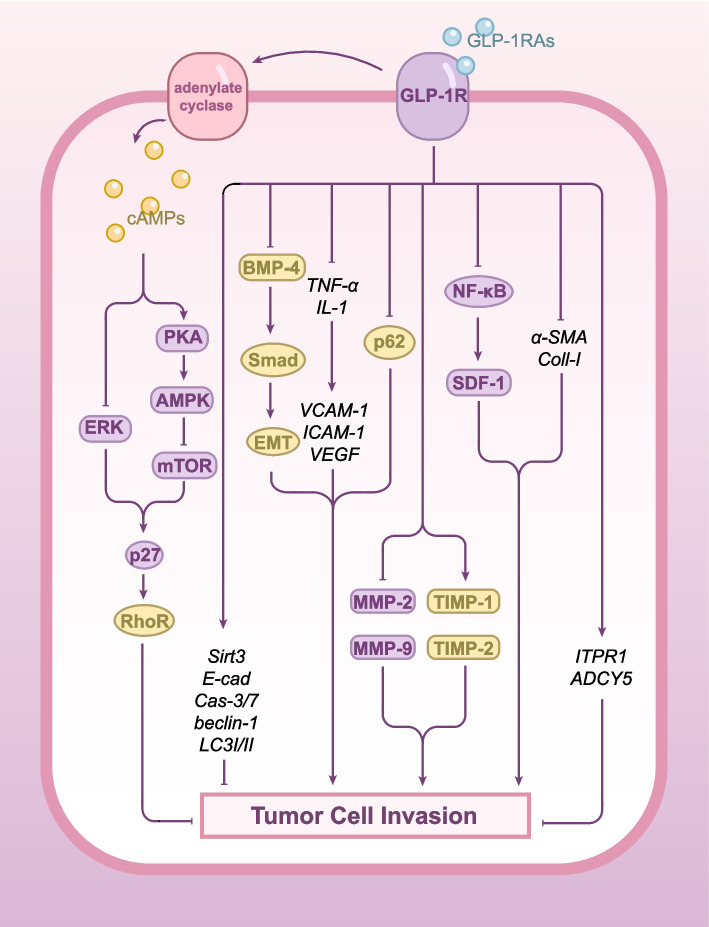
GLP-1RAs' Regulation of Tumor Cell Migration and Invasion. GLP-1RAs inhibit tumor cell migration and invasion through multiple mechanisms: (1) mTOR pathway suppression and p27 activation, leading to RhoA pathway inhibition; (2) PI3K/Akt/mTOR pathway inhibition; (3) Autophagy induction via beclin-1 and LC3I/II regulation; (4) BMP4-Smad pathway suppression, reducing EMT; (5) Downregulation of pro-inflammatory cytokines and their targets; (6) MMP-2/9 inhibition and TIMP-1/2 upregulation; (7) Sirt3 upregulation; and (8) NF-κB-SDF-1 axis suppression. Additional mechanisms include caspase-3/7 activation and regulation of ECM-related genes

GLP-1RAs exert their biological effects primarily by binding specifically to and activating the GLP-1 receptor (GLP-1R) [17, 38, 55]. As a Class B G-protein-coupled receptor (GPCR), GLP-1R regulates tumorigenesis through multiple signaling pathways. Its primary mechanisms involve the inhibition of tumor cell proliferation (Fig. 1), induction of tumor cell apoptosis (Fig. 2), and suppression of tumor cell migration and invasion.

#### The inhibition of cancer cell growth by GLP-1RAs through regulating cAMP-dependent signaling

GLP-1 receptor (GLP-1R) activation leads to adenylyl cyclase activation, subsequently inducing a substantial increase in intracellular cyclic adenosine monophosphate (cAMP) levels. Elevated cAMP acts mainly through the following molecular pathways: inhibition of the ERK pathway, activation of the PKA-AMPK-mTOR pathway, and activation of the p38 signaling pathway [13, 17, 56–58]: first, it inhibits the extracellular signal-regulated kinase (ERK) pathway, decreasing its activation of cyclin D1, thereby hindering cyclin D1-mediated DNA replication initiation and transition into the S phase of the cell cycle [17]. Additionally, Ex-4 has been demonstrated to reduce extracellular signal-regulated kinase (ERK)-mitogen-activated protein kinases (MAPK) phosphorylation in LNCaP prostate cancer cells [17]. This ERK-MAPK inhibition is closely associated with suppressed colon cancer cell proliferation [59]. Secondly, elevated cAMP levels activate protein kinase A (PKA), which subsequently mediates the activation of AMP-activated protein kinase (AMPK). AMPK antagonizes mammalian target of rapamycin (mTOR) activity, and its downstream effector, p27, inhibits cyclin-dependent kinase 2 (CDK2), thereby blocking the G1/S transition in the cell cycle, halting cell division, and subsequently inhibiting cellular proliferation [57]. Finally, elevated cAMP concentrations, in combination with Ex-4, activate the p38 signaling pathway in breast cancer cells, resulting in decreased cell growth and increased apoptosis [58]. Similarly, liraglutide induces cell growth inhibition and apoptosis in LNCaP prostate cancer cell lines via activation of the p38 signaling pathway [13].

cAMP-response element binding protein (CREB), a crucial downstream target of PKA, effectively suppresses breast cancer cell proliferation following GLP-1R activation [60, 61]. Ex-4 has been demonstrated to inhibit CT26 colon cancer cell proliferation through cAMP-PKA pathway-mediated Glycogen Synthase Kinase 3 (GSK3) inactivation [62].

#### The inhibition of cancer cell growth by GLP-1RAs through regulating PI3K/Akt signaling

Liraglutide suppresses protein kinase B (Akt) phosphorylation in pancreatic cancer cells by activating GLP-1R, thus inhibiting tumor growth via suppression of the PI3K/Akt signaling pathway [63]. It effectively suppresses the proliferation and migration of thyroid and liver cancer cells via inhibition of the PI3K/Akt/mTOR signaling pathway [59]. In liver cancer cells, liraglutide-mediated inhibition of this pathway elevates intracellular transforming growth factor-β1 (TGF-β1) levels, which induces cell cycle arrest, autophagy, and senescence [64]. Ex-4 has similarly been shown to inhibit the proliferation of prostate and breast cancer cells via suppression of the same pathway [65].

#### Additional molecular regulatory mechanisms

The inhibitory effect of GLP-1RAs on NF-κB has been shown to suppress the proliferation of pancreatic cancer cells [66]. Both in vitro and in vivo studies have provided evidence that Ex-4 can significantly inhibit breast cancer cell proliferation by suppressing NF-κB signaling activation and the expression of its downstream target genes, vascular endothelial growth factor (VEGF) and interleukin-8(IL-8) [55]. In vivo, Ex-4 has been demonstrated to inhibit prostate cancer cell growth by activating GLP-1R, reducing s-phase kinase-associated protein-2 (SKP2) gene expression, subsequently increasing p27Kip1 (p27) protein levels, and inducing tumor cell cycle arrest [67]. In endometrial cancer cells, liraglutide induces an upregulation in progesterone receptor (PGR) mRNA levels, promotes its protein expression, markedly enhances phosphorylated AMPK levels, and downregulates its downstream effector molecule p-P71S6K, ultimately inhibiting cell viability [58]. Moreover, Ex-4 induces autophagy and attenuates ovarian cancer cell viability by upregulating the expression of autophagic markers beclin-1 and microtubule-associated proteins light chain 3I/II (LC3I/II), while simultaneously downregulating the expression of p62 (a linker protein for LC3II) [68]. The Ex-4-induced autophagy is associated with reductions in cancer cell proliferation, enhanced apoptosis, and decreased invasion ability [68]. Apart from this, the reduction in matrix metalloproteinase-1 (MMP-1) levels mediated by GLP-1RA attenuates the proliferative capacity of cancer cells. GLP-1 analogs have been shown to additionally inhibit the growth of ovarian cancer cells by suppressing Akt phosphorylation [69].

The main mechanisms by which GLP—1RAs promote tumor apoptosis encompass the cAMP—PKA—dependent pathway, the BMP4/Smad signaling pathway, the PI3K/Akt/mTOR signaling pathway, and the NF-κB signaling pathway. The following are detailed descriptions of their signal cascade reactions [68, 70–72].

In addition to acting through the p38 pathway, GLP-1RAs also promote the increase of PKA levels via the cAMP-dependent pathway, which in turn prevents the phosphorylation of epidermal growth factor receptor (EGFR) and activator of transcription 3 (STAT3), resulting in the inactivation of the EGFR-STAT3 signaling pathway in tumor cells and subsequently downregulating several downstream effector genes such as myelocytomatosis oncogene (c-Myc), survivin, cyclin D1, Bcl-xl, and Bcl-2, thereby inducing apoptosis in tumor cells in a dose-dependent manner [70, 73].

Liraglutide, a member of the GLP-1RAs family, has been demonstrated to downregulate bone morphogenetic protein 4 (BMP4) expression in colorectal cancer (CRC) cells. As a member of TGF-β superfamily, BMP4 promotes epithelial-mesenchymal transition (EMT) via the canonical Smad signaling pathway, thereby driving tumor cell metastasis and invasion. Furthermore, liraglutide induces the activation of cleaved caspase-3 and downregulates Bcl-2 expression, implying that GLP-1RAs-mediated downregulation of BMP4 may promote apoptosis in CRC cells [71].

Relevant preclinical studies have demonstrated that Ex-4, by acting on GLP-1R, inhibits the PI3K/Akt signaling pathway and increases the expression of Caspase-3 and E-cadherin, thereby promoting apoptosis in human ovarian cancer cells and reducing their metastasis [72]. However, as GLP-1R expression is generally absent in most tumor specimens, the anticancer effects of Ex-4 may be restricted to specific patients with ovarian cancer who also have diabetes [72]. Within the cellular signaling network, Akt promotes mTOR activation, whereas AMPK functions as a negative regulator of mTOR. Ex-4 inhibits mTOR, an autophagy inhibitor in tumor cells, by downregulating Akt and simultaneously activating AMPK, subsequently attenuating the phosphorylation of its major downstream targets, eukaryotic translation initiation factor 4e binding protein 1(4E-BP1) and ribosomal s6 kinase 1(S6K1). These mechanisms result in the downregulation of Bcl-2 and NF-κB p65, alongside the upregulation of cleaved caspase-3/8 and Bax, ultimately inducing apoptosis in ovarian cancer cells [68].

NF-κB plays a crucial role as a key transcription factor in the regulation of tumor cell survival. NF-κB promotes tumor cell survival by upregulating a variety of anti-apoptotic proteins, including Bcl-2 family proteins and cell cycle regulators [68]. Previous studies demonstrated that Ex-4 inhibits breast cancer cell proliferation through NF-κB suppression, although significant apoptosis induction was not observed [55]. However, other studies have confirmed that Ex-4 induces apoptosis in breast cancer cells by regulating genes associated with cell survival and the extrinsic apoptotic pathway [74]. In ovarian cancer cell lines SKOV-3 and OVCAR-3, Ex-4 induces apoptosis by modulating NF-κB downstream targets, including matrix metalloproteinase-9 (MMP-9), intercellular adhesion molecule-1(ICAM-1), BAX, Bcl-2, and cyclin D [68]. Furthermore, liraglutide enhances the susceptibility of gemcitabine-resistant pancreatic cancer cells to apoptosis by downregulating ATP-binding cassette transporter G2 (ABCG2) via NF-κB signaling [75].

#### The inhibition of tumor cell migration and invasion by GLP1-RAs through regulating mTOR/p27/RhoA signaling pathway

In addition to the above—mentioned molecular mechanisms, GLP—1RAs also regulate the migration and invasion of tumor cells through the mTOR/p27/RhoA signaling pathway, the regulation of inflammatory cytokines and adhesion molecules, the matrix metalloproteinase system, and certain specific pathways. These mechanisms are specified below.

As previously mentioned, GLP-1RAs inhibit the mTOR signaling pathway, which in turn alleviates the inhibitory effect on the cell cycle regulatory protein p27 [17]. The p27 protein can inhibit the transduction of the rho-associated kinase (RhoA) signaling pathway, consequently reducing the migratory and invasive capacity of tumor cells [76]. Studies have demonstrated that liraglutide significantly impairs colorectal cancer cell migration, invasion, and proliferation by inhibiting the PI3K/Akt/mTOR signaling pathway, while concurrently promoting tumor cell apoptosis [77].

#### The inhibition of tumor cell migration and invasion by GLP1-RAs through regulating inflammatory factors and adhesion molecules, Matrix Metalloproteinase (MMP), and other molecules

Preclinical studies have demonstrated that Ex-4 exhibits anti-tumor effects in human ovarian cancer cell lines SKOV-3 and CAOV-3 by activating the GLP-1R [78]. Inflammatory cytokines in ovarian cancer cells, including tumor necrosis factor-α (TNF-α) and interleukin-1 (IL-1), promote malignant progression—such as tumor cell migration and invasion—by upregulating the expression of vascular cell adhesion molecule-1 (VCAM-1) and ICAM-1 [79–82]. Ex-4 significantly inhibits the metastatic potential of ovarian cancer cells by downregulating the expression of endothelial adhesion molecules and reducing the protein levels of vascular endothelial growth factor (VEGF) [78].

The balance between matrix metalloproteinases and their tissue inhibitors (TIMPs) is crucial in regulating ovarian cancer cell migration. Expression levels of matrix metalloproteinase-2 (MMP-2) and MMP-9 can be utilized as biomarkers to assess the metastatic potential of ovarian cancer and the invasiveness of breast cancer cells [83, 84]. Although the effects of MMPs on tumor cells vary depending on the specific MMP subtype, tumor type, and patient characteristics, studies indicate that decreased MMP expression is strongly associated with the inhibition of tumor cell metastasis [85, 86]. Research has demonstrated that GLP-1RAs significantly inhibit the expression of MMP-2 and MMP-9 in the human ovarian cancer cell line SKOV-3 within a TNF-α-induced inflammatory microenvironment, while simultaneously upregulating the levels of their inhibitors, tissue inhibitors-1 (TIMP-1) and tissue inhibitors-2 (TIMP-2) [78, 87].

In the SKOV-3 and CAOV-3 cell lines, Ex-4 induces the activation of caspase-3/7 (cas-3/7), leading to the inhibition of tumor cell migration and induction of apoptosis [78]. As a member of the mammalian sirtuins family, Sirt3 is a mitochondrion-localized histone deacetylase, which is highly expressed in mitochondria-rich tissues and plays a key regulatory role in the development of glioblastoma [87]. Preclinical studies have found that Ex-4 significantly upregulates Sirt3 expression in glioblastoma by activating the GLP-1R, which subsequently inhibits the growth, migration, invasion, and epithelial-mesenchymal transition of glioma cells [87]. Additionally, pancreatic cancer-related studies have shown that Ex-4 treatment reduces the mRNA expression levels of pancreatic stellate cell (PSC) activation markers α-SMA and Coll-I [39]. At the same time, Ex-4 inhibits the expression of NF-κB, subsequently reducing the expression and secretion of its downstream factor, stromal cell-derived factor-1 (SDF-1). The upregulation of SDF-1 plays a key role in PSC-mediated pancreatic cancer cell invasion [39]. In vivo experiments further confirmed that Ex-4 indirectly inhibits the proliferation and invasion of pancreatic cancer cells by suppressing PSC activity and extracellular matrix (ECM) deposition [39]. In CRC cells, GLP-1–related genes ITPR1 and ADCY5 may act as tumor suppressors [88]. Studies have shown that semaglutide upregulates the expression of ITPR1 and ADCY5 in CRC by activating the GLP-1 receptor, which, in turn, inhibits the motility of CRC cells [88].

#### Regulation of inflammatory and immune responses by GLP1-RAs

A substantial body of research suggests that the GLP-1 signaling pathway plays a pivotal role in regulating the infiltration of various anti-tumor immune cells, including CD4 + T cells, neutrophils, NK cells, macrophages, and dendritic cells. Among these, CD4 + T cells can crucially enhance the body’s anti-tumor immune response [16, 89, 90]. Macrophages and dendritic cells contribute to anti-tumor effects by curbing tumor progression, inducing apoptosis in tumor cells, and amplifying immune responses and immunosurveillance [88]. Preclinical studies has demonstrated that liraglutide enhances NK cell-mediated anti-tumor immune responses by inhibiting the IL-6/STAT3 signaling pathway in hepatocellular carcinoma cells and enhances NK cell functionality in obese patients [91]. Ex-4 is capable of suppressing the production of reactive oxygen species (ROS) in various cell types, with ROS playing a critical role in promoting the formation of tumor-associated neutrophil extracellular traps (NETs). Subsequent research demonstrated that the combination of Ex-4 and PD-1 inhibitor immunotherapy significantly enhances the body's anti-tumor immune response [92]. Similarly, liraglutide has been shown to enhance the efficacy of PD-1 inhibitors in the treatment of lung cancer and liver cancer [93].

Inflammation constitutes a critical factor in tumorigenesis and tumor progression. Numerous clinical studies have established that GLP-1RAs significantly modulate the body's inflammatory response. Multiple studies suggest that GLP-1RAs demonstrate significant anti-inflammatory properties across various cell and tissue types, effectively suppressing sustained inflammatory responses during chronic inflammation [94]. In a mouse model of influenza, the GLP-1 receptor agonist liraglutide not only inhibited the inflammatory response and improved survival rates but also significantly alleviated induced lung inflammation [94].

Non-alcoholic fatty liver disease (NAFLD) can progress to NASH, a major risk factor for liver cancer [95]. Animal and clinical studies have shown that GLP-1RAs ameliorate liver inflammation and lower the risk of NAFLD and NASH developing into liver cancer. They also reduce liver damage and inhibit tumor progression [43, 95]. The anti-inflammatory effects are often considered key mechanisms through which Ex-4 exerts its tumor-suppressive functions [96].

The novel GLP-1 receptor agonist (Ex-4)2-Fc, developed by Zhou et al., demonstrated anti-inflammatory activity in their study. Further studies revealed that (Ex-4)2-Fc inhibits NF-κB and p38 phosphorylation in adipose tissue while suppressing the MAPK signaling pathway. Experimental evidence demonstrated that treatment with (Ex-4)2-Fc promotes the conversion of pro-inflammatory M1 macrophages into anti-inflammatory M2 macrophages, leading to increased expression of anti-inflammatory factors and significant reductions in pro-inflammatory markers. Additionally, leptin expression in adipose tissue was suppressed, thereby inhibiting inflammatory responses in adipocytes via multiple regulatory mechanisms [97].

#### miRNA-mediated and epigenetic regulatory mechanisms

GLP-1RAs participate in the epigenetic regulation of the processes of tumorigenesis and cancer progression by modulating gene expression and changes in DNA methylation. Studies have demonstrated that GLP-1RAs promote AMPKα2 activation by inhibiting miR-27a expression in breast cancer cells. AMPKα2 plays a key role in tumor suppression by regulating cell cycle progression and apoptotic processes, which are mediated by cyclin D [96]. Moreover, in DNA methylation alterations of tumor cells induced by GLP-1RAs, studies have demonstrated that during breast cancer progression, tumor suppressor genes such as ESR1, CDH1, and ADAM33 are downregulated due to hypermethylation alterations in their promoter regions. Dulaglutide can restore the expression of these genes by inhibiting their methylation levels, thereby facilitating the reversion of tumor cells to their epithelial phenotype and suppressing tumor progression and invasive capabilities. This discovery provides a novel therapeutic strategy for overcoming breast cancer resistance and restoring sensitivity to estrogen therapy [98].

### The promotion of cancer development by GLP-1RAs

Several studies have reported a potential association between GLP-1RA use and increased thyroid cancer risk, although no causal relationship has been definitively established [1]. Prolonged GLP-1RAs therapy may exacerbate existing chronic inflammation in patients with T2DM, thereby potentially elevating their risk of pancreatic cancer. Observations from animal experiments and clinical case progression indicate that low-grade subclinical inflammation is sufficient for carcinogenesis. Furthermore, pancreatic ductal adenocarcinoma and pancreatic intraepithelial neoplasia, both associated with pancreatic cancer development, express GLP-1R, while normal pancreatic ductal cells and acini demonstrate proliferative responses to GLP-1RAs, suggesting that incretin-based medications may induce specific proliferative changes in the islets. Although the impact of GLP-1RAs on pancreatic cancer risk remains controversial, emerging evidence suggests that chronic low-grade inflammation and proliferative changes may constitute key pathophysiological mechanisms underlying pancreatic cancer development in patients receiving GLP-1RA therapy [8].

In addition, GLP-1RAs bind to GLP-1 receptors, activating the Akt signaling pathway, which subsequently enhances intracellular Wnt signal transduction [99]. Substantial evidence indicates that dysregulation of the Wnt/β-catenin signaling pathway is closely associated with tumorigenesis, particularly in colorectal cancer [100]. Although no current evidence suggests that GLP-1RAs induce β-catenin mutations or APC deficiencies, it is hypothesized that prolonged use of GLP-1RAs may foster the proliferative progression of colorectal cancer cells and pancreatic β-cells, based on their mechanisms of action [99].

Related studies have demonstrated that GLP-1R activation upregulates in situ expression of fibroblast growth factor-7 (FGF7) in intestinal tumor cells, promoting tumor progression and inducing intestinal hyperplasia. Additionally, FGF7 and its receptor fibroblast growth factor receptor-2 (FGFR2), along with its various isoforms, are significantly linked to the progression risk in breast cancer cell lines [18]. Considering the significantly elevated expression levels of GLP-1R in diabetic patients, it is hypothesized that administration of GLP-1R agonists may elevate the risk of breast cancer progression in these patients, thus necessitating particular caution in clinical management [101]. Evidence suggests that liraglutide may be implicated in breast cancer progression. In non-invasive MCF breast cancer cells, liraglutide has been shown to inhibit cellular growth [58]. However, in highly invasive breast cancer cell lines MDA-MB-231 and MDA-MB-468, high concentrations (100 nM) of liraglutide promote breast cancer progression by activating GLP-1R via the NOX4/ROS/VEGF signaling pathway [18]. Notably, this pro-tumorigenic effect was absent at a concentration of 50 nM, suggesting that the carcinogenic potential of liraglutide is concentration-dependent [18].

## Comparative analysis of cancer risk among glucose-lowering agents

Current clinical evidence indicates that various classes of antidiabetic drugs show substantial variability in their association with cancer risk. Notably, among these, metformin has demonstrated a well-established antitumor effect. Thiazolidinediones have been associated with a reduced risk of certain cancers, although concerns over their safety continue to be debated. The influence of SGLT2 inhibitors, dipeptidyl peptidase IV (DPP-4) inhibitors, and sulfonylureas on cancer risk remains inconclusive. Moreover, α-glucosidase inhibitors, insulin, and its analogs have not demonstrated significant associations with cancer risk in current studies. This section systematically examines the characteristics of cancer risk associations across various antidiabetic drugs, aiming to elucidate the patterns and differences in how these agents modulate cancer risk (Table 2).

**Table 2:** Comparative analysis of cancer risk among different classes of glucose-lowering medications

Type of drug	Risk prediction	Types of cancer affected
Metformin	↓	Bladder Cancer; Lung Cancer; Pancreatic Cancer; Head and Neck Cancer; Esophageal Cancer; Liver Cancer; Colorectal Cancer; Endometrial Cancer; Ovarian Cancer; Breast Cancer
TZD	↓	Lung Cancer; Liver Cancer; Colorectal Cancer; Breast Cancer; potentially Prostate Cancer and Pancreatic Cancer (increased risk in some studies)
SGLT2 Inhibitors	?	Bladder Cancer; Breast Cancer; potentially Pancreatic Cancer and Prostate cancer
DPP-4 Inhibitors	?	Colorectal Cancer; Prostate Cancer; Lung Cancer; Liver Cancer; Breast Cancer; Cholangiocarcinoma (elevated risk in some studies)
Sulfonylureas	?	General Malignancies; Prostate Cancer
α-Glucosidase Inhibitors	?	Not specified
Insulin and Insulin Analogs	?	Breast Cancer

*Abbreviations*: *TZD* thiazolidinediones, *SGLT2* sodium-dependent glucose transporters 2, *DPP-4* dipeptidyl peptidase IV, ↓: risk reduction, ?: controversial effects or limited evidence

### Hypoglycemic agents with clear anti-tumor effects

#### Metformin

Metformin is among the most widely prescribed oral hypoglycemic agents in clinical practice. In addition to its crucial role in the management of diabetes, metformin shows promising clinical applications in anti-tumor therapy. Large-scale epidemiological studies have confirmed that metformin users have a significantly lower risk of developing various malignancies, including bladder, lung, prostate, pancreatic, head and neck, esophageal, liver, and colorectal cancers, compared to non-users [10]. Systematic studies further reveal that metformin confers significant preventive effects against endometrial, ovarian, and breast cancers [11, 102]. Clinical data analysis suggests that metformin use reduces the risk of malignancies by approximately 30% [10] and significantly decreases tumor-related mortality in patients with type 2 diabetes [103]. Mechanistically, metformin exerts its anti-tumor effects primarily through two pathways: first, by improving insulin resistance, thereby reducing circulating insulin and IGF-1 levels, it indirectly inhibits tumor growth; second, metformin directly induces cell-cycle arrest and apoptosis in tumor cells, consequently suppressing tumor progression [104]. Of particular significance, three large-scale meta-analyses examining the relationship between metformin and prostate cancer risk demonstrated that while metformin significantly improved the prognosis of prostate cancer patients, its use showed no significant association with prostate cancer incidence. These meta-analyses were more comprehensive, incorporating a larger number of studies, featuring more recent publications, and demonstrating greater statistical power compared to previous meta-analyses. The relationship between metformin and prostate cancer incidence warrants cautious interpretation, necessitating further investigation through randomized controlled trials [105, 106]. In conclusion, while the association between metformin and the incidence of certain cancers remains a subject of debate, metformin exhibits significant protective effects against multiple types of tumors, demonstrating efficacy in inhibiting tumor cell proliferation and progression, reducing cancer risk, and improving cancer-related outcomes [107].

#### Thiazolidinediones (TZD) drugs

Systematic reviews and meta-analyses have demonstrated that TZD drugs are associated with a reduction in lung cancer risk in patients with type 2 diabetes of approximately 20% [98], and has a preventive effect on prostate cancer [102]. Another systematic review suggests that TZD drugs are associated with a reduction in the risk of liver cancer, colorectal cancer, as well as breast cancer [107]. However, some studies have reported that rosiglitazone and pioglitazone, both TZD drugs, could be associated with an increased risk of prostate and pancreatic cancer [107].

### Antidiabetic drugs with controversial effects on cancer risk

#### SGLT2 inhibitors

The current body of research on the relationship between SGLT2 inhibitors and cancer risk remains inconclusive. Several studies have suggested that the use of SGLT2 inhibitors does not significantly correlate with an elevated cancer risk [106, 107]. Research has indicated that the SGLT2 inhibitor empagliflozin can suppress breast cancer cell proliferation by inducing cell membrane hyperpolarization and mitochondrial membrane destabilization [32]. Additionally, empagliflozin has exhibited inhibitory effects on diverse tumor types, including pancreatic and prostate cancers [32]. However, certain studies have failed to establish a preventive effect of SGLT2 inhibitors on prostate cancer [102]. Current evidence suggests that dapagliflozin may elevate the risk of bladder and breast cancers, whereas other SGLT2 inhibitors have not demonstrated such associations [10]. Studies in patients with type 2 diabetes and obesity have demonstrated that the use of SGLT2 inhibitors, particularly empagliflozin, not only increases overall cancer risk but also significantly elevates the risk of bladder cancer [10, 108].

#### DPP-4 inhibitors

A systematic review of cardiovascular outcome trials (CVOTs) evaluating DPP-4 inhibitors demonstrated no elevated risk of pancreatic cancer or other malignancies [109]. Comprehensive meta-analyses of randomized controlled trials indicated no significant association between DPP-4 inhibitor therapy and overall cancer risk; notably, subgroup analyses of placebo-controlled trials demonstrated a significant reduction in both colorectal and overall cancer risk [10]. An analysis of the SEER database demonstrates that DPP-4 inhibitors may improve survival outcomes in patients with colorectal and lung cancer [19]. Clinical evidences on the DPP-4 inhibitor sitagliptin suggests it is associated with a decreased risk of prostate cancer [106, 110], and long-term administration of this agent may have potential benefits for breast cancer [96]. Mechanistic insights reveal that sitagliptin exerts anti-tumor activity by augmenting eosinophil-mediated immune responses in preclinical orthotopic models of hepatocellular carcinoma and breast cancer [96]. In vitro studies confirm that linagliptin suppresses the proliferation of several tumor cell lines, including colorectal cancer HCT116, breast cancer MCF-7, and liver cancer HepG2, via mechanisms that involve cell cycle arrest and apoptosis induction [96]. Preclinical studies have demonstrated that DPP-4 inhibitors significantly prevent the onset of liver cancer in MC4R-KO mice [111]. Separate analytical study examining 222 exposure events revealed no statistically significant correlation between DPP-4 inhibitor administration and cholangiocarcinoma risk [112] Conversely, a Large-scale population-based cohort studies demonstrated that DPP-4 inhibitor utilization, when compared with other second- or third-line glucose-lowering agents, was associated with an elevated risk of cholangiocarcinoma (HR = 1.77, P < 0.05) [112]. Compared to other second- or third-line antidiabetic agents, DPP-4 inhibitors have not been shown to increase the risk of lung cancer [48]. And the use of DPP-4 inhibitors did not raise the risk of thyroid cancer compared with SGLT2 inhibitors or other treatment alternatives [113, 114].

#### Sulfonylureas

Current evidence demonstrates significant heterogeneity in the effects of sulfonylureas on cancer risk. Epidemiological evidence suggests that sulfonylurea use is associated with an approximately 20% increased risk of specific malignancies, although the causality of this association requires further validation due to current evidence limitations [10]. From a mechanistic perspective, sulfonylureas may facilitate tumor formation and progression via their insulin secretagogue properties [102]. Systematic meta-analyses have established that sulfonylurea therapy shows no protective effect against prostate cancer development in patients with diabetes mellitus [102]. Clinical evidences have demonstrated that glibenclamide, a prototypical sulfonylurea, is associated with a significantly increased cancer risk occurrence [107]. However, Several preliminary studies have suggested that sulfonylureas might exert protective effects against specific types of cancer [107].

### Antidiabetic drugs with limited evidence for tumor risk association

#### α-Glucosidase inhibitors

As an antidiabetic drug, α-glucosidase inhibitors primarily act by inhibiting the breakdown of complex carbohydrates in the gastrointestinal tract, thereby slowing their absorption and mitigating postprandial hyperglycemia. Moreover, α-glucosidase inhibitors can elevate plasma GLP-1 levels. In general, the use of α-glucosidase inhibitors is associated with mild weight loss, potentially due to their effects on nutrient absorption and appetite regulation. Nonetheless, current clinical studies have not identified a clear association between the use of these medications and elevated oncogenic risk [107].

#### Insulin and insulin analogs: cancer risk assessment

As one of the most effective anti-diabetic therapies currently available, insulin therapy for glycemic control carries risks of hypoglycemia and weight gain. Additionally, several studies have explored the cancer risks associated with insulin analogs and their comparison to human insulin treatments. Some studies—which have faced methodological critiques—suggest that insulin glargine may increase the risk of breast cancer. A systematic review of data from 265 studies has indicated that no significant association exists between any form of insulin therapy and an increased cancer risk. Furthermore, a multinational cohort study involving five countries revealed that neither insulin detemir nor insulin glargine significantly increases cancer risk compared to human insulin [107].

## Modifying factors in the association between GLP-1RAs and cancer risk

The section primarily reviews the various factors influencing the cancer risk associated with GLP-1RAs, including patient characteristics, comorbidities, clinical treatment, and lifestyle. In terms of patient characteristics, the reduction in prostate cancer risk with GLP-1RAs is more significant in older male patients, and the expression of endogenous GLP-1 receptors has been linked to cancer risk. Regarding ethnicity and genetic background, different ethnic groups exhibit differential effectiveness of GLP-1RAs in the reduction of lung cancer risk. For comorbid metabolic diseases, such as diabetes and obesity, the use of GLP-1RAs generally does not increase cancer risk and may offer certain benefits. In patients with cardiovascular diseases, clinical observations have demonstrated enhanced benefits of GLP-1RAs in treating prostate cancer. Additionally, factors related to clinical treatment—such as the type of GLP-1RAs, dosage, combination therapy, and duration of treatment—have exhibited diverse impacts with respect to cancer risk. Finally, patient lifestyle factors, such as smoking and alcohol consumption, are also linked to the modulation of cancer risk by GLP-1RAs.

### Patient characteristics affecting the tumor risk of GLP-1RAs

#### Age and gender

Meta-analyses have demonstrated that GLP-1RAs reduce the risk of prostate cancer, particularly in older male populations [115]. Prospective studies have suggested that elevated endogenous GLP-1 receptor expression may be associated with a reduced risk of initial cancer onset. Research has revealed that after adjusting for age and gender, endogenous GLP-1 receptor expression was weakly correlated with fasting blood glucose levels and an analogous increase in cancer risk. However, this relationship diminished considerably after additional adjustments for these demographic factors [6]. Furthermore, these studies have identified a threshold effect in male subjects, wherein fasting GLP-1 levels appeared to confer a protective effect against cancer risk [6].

#### Racial and genomic determinants

In a nationwide retrospective cohort study, Tabernacki et al. demonstrated that GLP-1RA administration significantly reduced lung cancer risk in Black and White populations (P < 0.05). However, similar protective effects were not observed in other racial groups or Hispanic populations, potentially attributable to limited statistical power in these subgroup analyses [19]. The American Heart Association's Heart Disease and Stroke Statistics report has documented significant racial disparities in comorbidity management outcomes and healthcare delivery [116]. The possible reasons are substantial heterogeneity exists in healthcare accessibility and comorbidity management across ethnic populations, contributing to differential health outcomes. Notably, epidemiological data indicate that Asian populations develop diabetes at a significantly younger age than White populations, exhibiting elevated risks of disease-related complications and mortality, thereby imposing a considerable healthcare burden [117]. Distinct disease risk profiles have been documented among Black and Hispanic populations, reflecting unique pathophysiological patterns [118, 119].

### Coexisting conditions affecting the tumor risk of GLP-1RAs

#### Metabolic diseases

Large-scale clinical studies have confirmed that the use of GLP-1RAs in diabetic populations does not increase the cancer risk occurrence. Preclinical in vitro and in vivo studies indicate that GLP-1RA treatment does not exacerbate cancer risk in patients with type 2 diabetes and concurrent pancreatic cancer [13]. Clinical evidence further confirmed Liraglutide significantly reduces the mortality risk in patients with type 2 diabetes and concurrent prostate cancer [44]. Research has demonstrated that, in comparative studies of anti-diabetic drugs, GLP-1RAs can significantly reduce the risk of liver decompensation and hepatocellular carcinoma in patients with type 2 diabetes [42]. In type 2 diabetes-related cervical cancer tissues, a high-glucose microenvironment induces the upregulation of PSMA2, phosphorylated IκB, and phosphorylated P65 expression, thereby promoting tumor progression, while Ex-4 can effectively inhibit this pathological process [120]. Animal model studies suggest that liraglutide significantly inhibits liver cancer occurrence in diabetic mouse models, and this inhibitory effect may be partly attributed to its ability to improve the high-glucose microenvironment [44].

Emerging clinical evidence suggests that GLP-1-mediated insulin secretion regulation is impaired in conditions of obesity and insulin resistance [121]. GLP-1RAs attenuate the progression of NASH to hepatocellular carcinoma through enhancement of hepatic metabolic function. Moreover, epidemiological data have established a significant association between visceral obesity and the pathogenesis of primary hepatic malignancies [15]. Clinical investigations demonstrate that GLP-1RA therapy exhibits superior therapeutic efficacy in patients without hepatic steatosis compared to those with fatty liver disease [42]. Preclinical studies have demonstrated that liraglutide significantly suppresses obesity-associated breast cancer cell proliferation via modulation of adipokine expression, with enhanced antineoplastic effects observed in obese populations [122]. Recent clinical trials reveal that despite semaglutide-induced significant weight reduction, this therapeutic effect has not corresponded to a decreased cancer risk [123].

#### Other comorbidities

Various studies have demonstrated significant cardiovascular benefits of GLP-1RAs [2], and current clinical guidelines recommend their use as adjunctive therapies in the management of cardiovascular diseases [22]. Relevant meta-analyses suggest that GLP-1RA treatment exerts more pronounced antitumor effects in prostate cancer, particularly in individuals with coexisting cardiovascular diseases [115]. Furthermore, a meta-analysis of data from large cardiovascular outcome trials (CVOT) demonstrates that GLP-1RA therapy does not seem to elevate the risk of pancreatic cancer in patients with T2DM [7].

In a murine model, cervical undifferentiated carcinoma cells expressing human papillomavirus type 16 E7 protein (HPV-16E7) contribute to cervical carcinogenesis via immunomodulatory mechanisms. Conversely, Ex-4, as a GLP-1 receptor agonist (GLP-1RA), demonstrates potent inhibitory effects on these tumor cells [96].

### Pharmacological determinants in GLP-1RA-Associated cancer risk

#### Differential cancer risk profiles across GLP-1RA subtypes and dosing regimens

Existing studies underscore significant differences in the effect of various GLP-1RAs on the risk of specific cancers, such as pancreatic cancer [6–8, 26, 27]. This finding highlights the necessity for meticulous consideration of specific GLP-1RA types in clinical decision-making. Additionally, GLP-1RA dosage exhibits a dose-dependent effect on cancer risk modulation. Research has demonstrated that higher concentrations of liraglutide may exhibit tumor-promoting effects [18]. In contrast, Ex-4 has exhibited significant anti-tumor proliferation effects within a concentration range of 0.5 to 10 nM; however, this inhibitory effect diminishes when concentrations increase to 50–100 nM [60].

#### The modulatory effects of combined therapy with GLP-1RAs and other drugs on tumor risk

As GLP-1RAs continue to see expanding clinical application, it is imperative that clinicians give careful consideration to the potential adverse effects linked to combination therapies. Several case reports indicate that the concurrent use of GLP-1RAs and dipeptidyl peptidase-IV inhibitors (DPP-4Is) might elevate the risk of developing specific cancers, such as breast, thyroid, and pancreatic cancers [124]. This heightened risk might be associated with the exacerbation of chronic inflammatory states in patients undergoing prolonged combination therapy, potentially facilitating carcinogenic processes [8].

#### Combined therapeutic strategy of GLP-1RAs and antidiabetic medications

The combination of SGLT2 inhibitors or thiazolidinediones with GLP-1RAs has been shown to significantly enhance therapeutic efficacy against liver cancer. However, it should be noted that this combination therapy strategy does not reverse the tumor-promoting effects of previous insulin treatments [42]. Additionally, a potential synergistic mechanism may exist between SGLT2 inhibitors and GLP-1RAs in the management of NASH [42]. Regarding antitumor activity, the combination of GLP-1RAs and metformin has shown substantial clinical benefit. Exenatide, when combined with metformin, has been shown to significantly suppress the proliferation of breast cancer cells [104]. Liraglutide, in combination with metformin, has demonstrated synergistic antipancreatic cancer effects in both in vivo and in vitro studies [8].

#### Therapeutic synergy between GLP-1RAs and antineoplastic agents

The combination of GLP-1RAs with various antineoplastic agents not only demonstrates significant tumor-suppressive effects but also has the potential to augment the clinical outcomes of current treatment regimens. The combination of a levonorgestrel-releasing intrauterine device (LNG-IUD), metformin, and liraglutide has been shown to safely and effectively improve endometrial hyperplasia in obese patients, as well as promote the regression of early-stage endometrial cancer [41]. Preclinical studies have demonstrated that the combination of liraglutide and medroxyprogesterone acetate produces a significantly stronger inhibitory effect on endometrial cancer cell proliferation compared to monotherapy, and this effect exhibits dose-dependency [58]. In prostate cancer treatment, the combination of liraglutide and docetaxel has been demonstrated to significantly improve therapeutic outcomes. The combined actions of these two agents increase Bax expression, inhibit Bcl-2 levels, and downregulate the phosphorylation of ERK and Akt, collectively suppressing prostate cancer progression and promoting apoptosis. Ex-4 may also mitigate enzalutamide (ENZ) resistance and synergistically inhibit prostate cancer cell proliferation, potentially by inhibiting ENZ-induced Akt activation [69].

#### Temporal impact of treatment duration on tumor risk

The duration of treatment with GLP-1RAs is a critical factor influencing clinical outcomes. Clinical evidence indicates that treatment with GLP-1RAs for durations of 1 to 3 years or longer may be associated with an elevated risk of thyroid carcinoma, particularly medullary thyroid carcinoma [4]. However, long-term studies on GLP-1RAs exposure indicate no significant changes in the risks of thyroid, bladder, or pancreatic cancers, whereas the incidence of prostate, lung, and colorectal cancers has been shown to significantly decrease [20]. In addition, the pro-carcinogenic effects of liraglutide on two highly aggressive breast cancer cell lines, MDA-MB-231 and MDA-MB-468, were most pronounced after 2 to 3 years of continuous exposure [18].

Studies on the long-term safety of GLP-1RAs suggest temporal and individualized variations in their clinical application. In the treatment of obesity, prolonged use of semaglutide may result in tolerance and subsequent weight regain following discontinuation [125]. GLP-1RAs have demonstrated optimal clinical efficacy when administered as a therapeutic intervention during the early stages of tumor development [36]. This observation emphasizes not only the importance of proper treatment timing but also offers critical insights for clinical practice. Based on this evidence, it is recommended that long-term pharmacological management prioritize the optimization of treatment timing, formulation of personalized dosing regimens, and implementation of systematic efficacy monitoring and adverse reaction assessment protocols, to maximize therapeutic benefits while minimizing potential risks.

### Lifestyle factors affecting the association between GLP-1RAs and cancer risk

Smoking status is a primary determinant in the alteration of lung cancer risk. Among individuals who have never smoked, the use of incretin-related drugs does not appear to be associated with altered lung cancer risk [48]. However, other studies have indicated that GLP-1RAs are linked to a reduced risk of lung cancer, independent of smoking status [19]. GLP-1RAs have been correlated with a decreased risk of hepatic decompensation and liver cancer, with this protective effect being more significant in patients who do not have alcohol or tobacco use disorders as compared to those who do [42].

## Conclusion

**Fig. 3:**
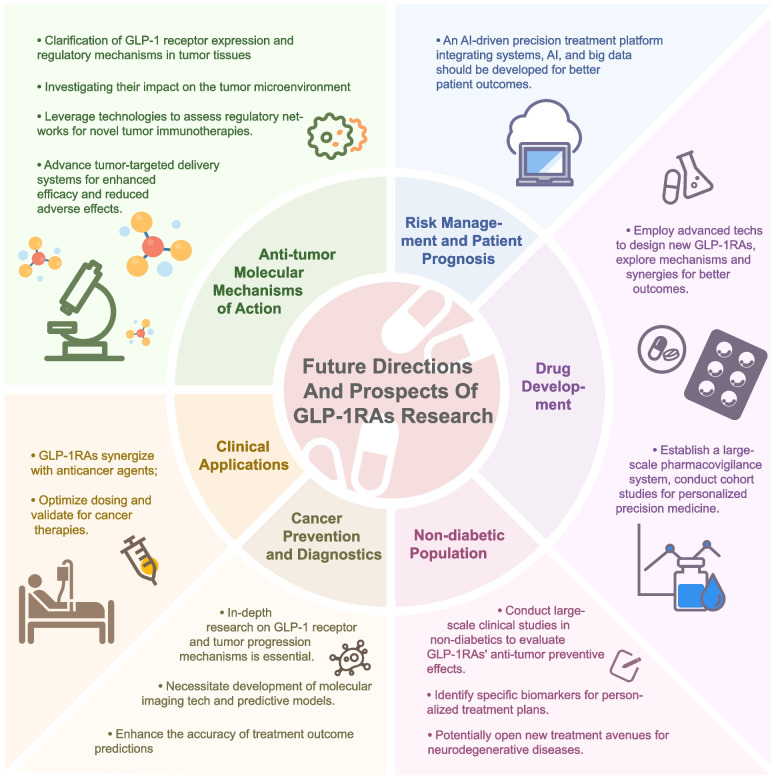
Future Research Directions for GLP-1RAs in Cancer Treatment. This figure outlines key research priorities: (1) Molecular mechanism elucidation, including receptor expression patterns and tumor microenvironment interactions; (2) Clinical application optimization, focusing on combination therapies and precision medicine approaches; (3) Prevention and diagnostic developments, emphasizing biomarker identification and predictive modeling; (4) Drug development advances, including design of selective analogs and multi-target compounds; and (5) Establishment of comprehensive pharmacovigilance systems incorporating AI-driven precision medicine platforms for improved patient outcomes

This comprehensive review systematically investigates the intricate relationship between GLP-1RAs and cancer risk. The evidence reveals significant heterogeneity in the effects of GLP-1RAs across different cancer types. Current evidence indicates protective effects against several malignancies (including colorectal, hepatic, and prostate cancers), whereas other studies suggest potential risks for specific cancer types, particularly thyroid cancer. These diverse effects are mediated through multiple molecular mechanisms, including metabolic regulation, direct antitumor activities, immunomodulation, and epigenetic modifications. The molecular mechanisms by which GLP-1RAs influence cancer risk involve complex intracellular signaling networks. These pathways include the cyclic AMP-protein kinase A (cAMP-PKA) pathway, phosphatidylinositol 3-kinase/protein kinase B/mammalian target of rapamycin (PI3K/Akt/mTOR) signaling, and nuclear factor kappa B (NF-κB)-mediated cascades, which regulate cell proliferation, apoptosis, and migration. The immunomodulatory effects of GLP-1RAs, specifically their impact on antitumor immune responses and inflammatory signaling pathways, constitute a critical mechanism influencing cancer risk. Comparative analyses of GLP-1RAs with other glucose-lowering agents reveal distinct safety profiles, emphasizing the critical importance of patient-specific treatment strategies. The potential relationship between GLP-1RAs and cancer risk is modulated by diverse clinical factors, encompassing patient demographics, comorbidities, treatment protocols, and lifestyle interventions. Moving forward, several critical research directions warrant investigation (Fig. 3): (1) comprehensive elucidation of molecular mechanisms underlying the antitumor effects, specifically utilizing advanced methodologies including single-cell sequencing and multi-omics approaches [18, 88, 89, 91, 92, 126]; (2) optimization of combination therapy strategies with established anticancer agents [13, 18, 31, 33, 97, 127–129]; (3) development of next-generation GLP-1 receptor-targeted therapeutics with enhanced specificity and reduced off-target effects [129–132]; and (4) establishment of sophisticated pharmacovigilance networks incorporating artificial intelligence algorithms to optimize therapeutic decision-making processes. These advances will be crucial for enhancing our understanding of GLP-1RAs in cancer prevention and treatment, ultimately advancing the paradigm of precision medicine in this field.

## Data Availability

No datasets were generated or analysed during the current study.

## Abbreviations

ABCG2: ATP-binding cassette transporter G2
Akt: Protein kinase B
AMPK: AMP-activated protein kinase
BMP4: Bone morphogenetic protein 4
cAMP: Cyclic adenosine monophosphate
cas-3: Caspase-3
CDK2: Cyclin-dependent kinase 2
c-Myc: Myelocytomatosis oncogene
CRC: Colorectal cancer
CREB: CAMP-response element binding protein
DPP-4: Dipeptidyl peptidase IV
E-cad: E-cadherin
ECM: Extracellular matrix
EGFR: Epidermal growth factor receptor
ENZ: Enzalutamide
EMT: Epithelial-mesenchymal transition
ERK: Extracellular signal-regulated kinase
Ex-4: Exendin-4
FGFR: Fibroblast growth factor receptor
GIP: Glucose-dependent Insulinotropic Polypeptide
GLP-1: Glucagon-like peptide-1
GLP-1RAs: Glucagon-like peptide-1 receptor agonists
GPCR: G-protein-coupled receptor
GSK3: Glycogen Synthase Kinase 3
HPV-16E7: Human papillomavirus type 16 E7 protein
ICAM-1: Intercellular adhesion molecule-1
IGF: Insulin-like growth factor
IL: Interleukin
LC3I/II: Microtubule-associated proteins light chain 3I/II
LNG-IUD: Levonorgestrel-releasing intrauterine device
MAPK: Mitogen-activated protein kinases
MMP: Matrix metalloproteinase
mTOR: Mammalian target of rapamycin
NAFLD: Non-alcoholic fatty liver disease
NASH: Non-alcoholic steatohepatitis
NETs: Neutrophil extracellular traps
NF-κB: Nuclear factor kappa B
PGR: Progesterone receptor
PI3K: Phosphatidylinositol-4,5-bisphosphate 3-kinase
PKA: Protein kinase A
PSC: Pancreatic stellate cell
RCTs: Randomized controlled trials
RhoA: Rho-associated kinase
ROS: Reactive oxygen species
S6K1: Ribosomal s6 kinase 1
SDF-1: Stromal cell-derived factor-1
SGLT2: Sodium-dependent glucose transporters 2
SKP2: S-phase kinase-associated protein-2
STAT3: Signal transducer and activator of transcription 3
T2DM: Type 2 diabetes mellitus
TGF-β: Transforming growth factor-beta
TIMP: Tissue inhibitor of metalloproteinase
TNF-α: Tumor necrosis factor-alpha
TZD: Thiazolidinediones
VCAM-1: Vascular cell adhesion molecule-1
VEGF: Vascular endothelial growth factor
